# Trehalose-Mediated Autophagy Impairs the Anti-Viral Function of Human Primary Airway Epithelial Cells

**DOI:** 10.1371/journal.pone.0124524

**Published:** 2015-04-16

**Authors:** Qun Wu, Di Jiang, Chunjian Huang, Linda F. van Dyk, Liwu Li, Hong Wei Chu

**Affiliations:** 1 Department of Medicine, National Jewish Health, Denver, Colorado, United States of America; 2 Department of Immunology and Microbiology, University of Colorado Denver School of Medicine, Aurora, Colorado, United States of America; 3 Department of Biological Sciences, Virginia Polytechnic Institute and State University, Blacksburg, Virginia, United States of America; Central Michigan University School of Medicine, UNITED STATES

## Abstract

Human rhinovirus (HRV) is the most common cause of acute exacerbations of chronic lung diseases including asthma. Impaired anti-viral IFN-λ1 production and increased HRV replication in human asthmatic airway epithelial cells may be one of the underlying mechanisms leading to asthma exacerbations. Increased autophagy has been shown in asthmatic airway epithelium, but the role of autophagy in anti-HRV response remains uncertain. Trehalose, a natural glucose disaccharide, has been recognized as an effective autophagy inducer in mammalian cells. In the current study, we used trehalose to induce autophagy in normal human primary airway epithelial cells in order to determine if autophagy directly regulates the anti-viral response against HRV. We found that trehalose-induced autophagy significantly impaired IFN-λ1 expression and increased HRV-16 load. Inhibition of autophagy via knockdown of autophagy-related gene 5 (ATG5) effectively rescued the impaired IFN-λ1 expression by trehalose and subsequently reduced HRV-16 load. Mechanistically, ATG5 protein interacted with retinoic acid-inducible gene I (RIG-I) and IFN-β promoter stimulator 1 (IPS-1), two critical molecules involved in the expression of anti-viral interferons. Our results suggest that induction of autophagy in human primary airway epithelial cells inhibits the anti-viral IFN-λ1 expression and facilitates HRV infection. Intervention of excessive autophagy in chronic lung diseases may provide a novel approach to attenuate viral infections and associated disease exacerbations.

## Introduction

Human rhinovirus (HRV) is the most frequently detected respiratory virus in all age groups of human subjects who suffer from acute infections in the upper (e.g., common cold) as well as the lower (e.g., bronchiolitis and pneumonia) airways [1]. Most importantly, HRV is the major cause for acute exacerbations of chronic lung diseases such as asthma, chronic obstructive pulmonary diseases, and cystic fibrosis [1–3]. HRV belongs to the picornaviridae family with single stranded RNA, and has been categorized into major (e.g., HRV-16) and minor (e.g., HRV-1A and HRV-1B) groups that bind host cell intercellular adhesion molecule 1 and low-density lipoprotein receptor, respectively. Airway epithelial cells represent the primary site of HRV infection *in vivo* [4, 5]. Interestingly, recent studies suggest that IFN-λ1, a type III anti-viral interferon, is the major type of IFNs induced during HRV infection in human primary airway epithelial cells [6–8] and serves as a crucial anti-viral mechanism against HRV infection [9]. Impaired IFN-λ1 production and increased HRV-16 replication have been reported in cultured human airway epithelial cells from asthmatics [10]. However, the exact mechanisms underlying the impaired anti-viral interferon (i.e., IFN-λ1) response have not been well elucidated.

Autophagy is an essential homeostatic pathway by which cells degrade damaged or obsolete organelles and proteins through the lysosomal machinery [11, 12]. There is evidence of increased autophagy in airway epithelial cells of asthmatics [13, 14], but the function of autophagy in human airway epithelium, especially in the context of asthma-related viral (e.g., HRV) infection, has not been explored. Recent studies suggest that autophagy serves as a novel host defense mechanism against viral infections [15]. But, the interplay between autophagy and anti-viral interferon response during viral infections is complex. Production of type I IFN-α in response to infection of some RNA viruses (e.g., hepatitis C virus and HIV-1) depends on the autophagic pathway [16, 17]. In contrast, the activation of autophagic pathway during infection of certain RNA viruses (e.g., vesicular stomatitis virus, herpesvirus and hepatitis C virus) appears to block the production of type I IFN-β [18–20] and thereby promotes viral replication. Mechanistically, the autophagy-related gene 5 (ATG5)-ATG12 conjugate, a key regulator of the early autophagic process, may interact with retinoic acid-inducible gene I (RIG-I) and IFN-β promoter stimulator 1 (IPS-1) to negatively regulate the expression of type I IFN-β [18, 21]. So far, whether ATG5 regulates the expression of type III interferons, especially IFN-λ1, in HRV-infected human airway epithelial cells remains unclear.

Trehalose is a natural glucose disaccharide found across the three domains of life and has multiple biological functions such as preventing LPS-mediated inflammatory response [22, 23]. Recently, trehalose has been recognized as an effective autophagy inducer in various mammalian cells [24, 25]. Trehalose induces autophagy by promoting the recruitment of LC3 II, the conjugated form of LC3 I with phosphatidylethanolamine (PE), into the forming autophagosome membrane in an ATG5-ATG12-dependent manner [18]. Thus, trehalose-induced autophagy serves as an excellent model to directly dissect the role of autophagy in regulating the anti-viral (e.g., HRV) response in human airway epithelial cells.

In the present study, we hypothesized that induction of autophagy inhibits anti-viral IFN-λ1 response and subsequently promotes HRV-16 infection in human airway epithelial cells. We first examined the effects of trehalose on IFN-λ1 expression and HRV-16 load in normal human primary airway epithelial cells. We then knocked down ATG5 gene to determine the role of trehalose-induced autophagy in inhibiting airway epithelial anti-viral responses. Lastly, to demonstrate the potential molecular mechanisms underlying autophagy-mediated inhibition of airway epithelial anti-viral function, we examined the interaction of ATG5 protein with RIG-I and IPS-1.

## Materials and Methods

### Preparation of HRV-16

HRV-16 (American Type Culture Collection, Manassas, VA) was propagated in H1-Hela cells (CRL-1958, ATCC), and purified as described previously [26]. H1-Hela cells are susceptible to rhinovirus infection and thus very useful for passaging and titrating rhinoviruses. Viral stocks were titrated by infecting H1-HeLa monolayers with serially diluted HRV-16 to assess the cytopathic effect, and the viral titer was expressed as 50% tissue culture infective doses per ml (TCID_50_/ml) [27].

### Trehalose treatment and HRV-16 infection in normal human primary airway epithelial cells

Normal human tracheobronchial epithelial (NHTE) cells from never smokers were isolated from the tracheas and bronchi of de-identified organ donors whose lungs were not suitable for transplantation as described previously [28]. We obtained the donor lungs through the International Institute for the Advancement of Medicine (Edison, NJ) and the National Disease Research Interchange (Philadelphia, PA). The collection of human tracheobronchial epithelial cells was approved by the institutional review board (IRB) of National Jewish Health. Briefly, cells at passage 2 were seeded into 12-well cell culture plates at 1 × 10^5^ cells/well in bronchial epithelial cell growth medium (BEGM) with supplements (Lonza, Walkersville, MD) at 37°C, 5% CO_2_. At 40–50% confluence, cells were treated with medium (control) or with 100 mM trehalose (Sigma-Aldrich, St. Louis, MO) for 48 h to induce autophagy. The concentration and pre-incubation period of trehalose were chosen based on previous publications [24, 29–31] and our preliminary study. Thereafter, medium- or trehalose-treated cells were infected with HRV-16 at 10^4^ TCID_50_/well or sterile PBS (mock infection control). Two hours later, cells were washed three times to remove free viruses and then cultured in BEGM with or without trehalose (100 mM) for additional 6 or 24 h to measure IFN-λ1 expression, HRV RNA levels, activity of lactate dehydrogenase (LDH) (a marker of cytotoxicity), or LC3 I and LC3 II proteins.

### ATG5 gene knockdown in normal human primary airway epithelial cells

NHTE cells at passage 2 were seeded at 2 × 10^5^ cells/well onto collagen-coated 12-well cell culture plates. ATG5 chimera siRNA (ATG5 siRNA, H00009474-R01, Abnova, Taipei, Taiwan) or Naito1 chimera RNAi (control siRNA, R0017, Abnova) was transfected into cells at 60–70% confluence using siRNA transfection reagents (Santa Cruz Biotechnology Inc., Santa Cruz, CA) according to the manufacturer's instructions. Twenty-four hours after siRNA transfection, cells were treated with or without 100 mM trehalose for 48 h to induce autophagy. Thereafter, cells were infected with HRV-16 at 10^4^ TCID_50_/well or sterile PBS (control) as described above. Cells were processed to examine ATG5 knockdown by Western blot, IFN-λ1 mRNA expression and HRV RNA levels by quantitative RT-PCR after 6 h of HRV-16 infection when the anti-viral response is expected to peak.

### Lactate dehydrogenase (LDH) assay

To quantitate the cytotoxic effects of trehalose and HRV-16 infection, cell culture supernatants were subjected to measure LDH levels using a cytotoxicity detection kit (Roche Diagnostics, Indianapolis, IN) according to the manufacturer’s instruction. The optical density values at 450 nm (OD450nm) were determined using a microplate reader.

### Western blot analysis

Equal amounts of protein samples from different treatments were separated on 10% or 15% SDS—PAGE, transferred onto polyvinylidene difluoride (PVDF) membranes, and probed with rabbit anti-LC3 (Sigma-Aldrich), rabbit anti-ATG5 antibody (Novus Biological, Littleton, CO), rabbit anti-RIG-I antibody (Cell Signaling Technology Inc., Danvers, MA), mouse anti-IPS-1 (Santa Cruz Biotechnology Inc.), or mouse anti-GAPDH (Santa Cruz Biotechnology Inc.). Blots were then incubated with appropriate HRP-linked secondary antibodies and ECL Western blotting substrate. Densitometry was performed using the NIH Image-J software. The ratios of LC3 II/LC3 I protein were used to indicate the formation of autophagosomes.

### Immunoprecipitation (IP)

NHTE cells at passage 2 were seeded at 5 × 10^5^/well onto collagen-coated 6-well cell culture plates and were treated with or without 100 mM trehalose for 48 h to induce autophagy. Thereafter, cells were infected with HRV-16 at 10^4^ TCID_50_/well or sterile PBS (control) as described above. After 6 h of HRV-16 infection, cells were lysed in IP lysis buffer consisting of 50 mM Tris-HCl (pH 8.0), 120 mM NaCl, 1% NP-40, 4 mM EDTA, 50 mM NaF, 1 mM Na_3_VO_4_, and 1× protease inhibitor cocktail. After the cell lysate was sonicated and centrifuged, the supernatant was transferred and pre-cleared with protein-G agarose beads (Santa Cruz Biotechnology Inc.) that contained the mouse isotype control IgG for 1 h at 4°C. The pre-cleared supernatant was incubated with 0.5 μg of mouse anti-human ATG5 antibody (clone ATG5-18, Sigma-Aldrich) at 4°C for 2 h on a rotator. Immunoprecipitated proteins were separated on 10% SDS—PAGE for Western blot analysis of RIG-I, IPS-1 and ATG5.

### Quantitative real-time RT-PCR

Taqman quantitative real-time RT-PCR was used to detect human IFN-λ1 mRNA expression and HRV RNA levels as previously described [32]. The specific primers and probes are: IFN-λ1 (forward: 5′-GGG AAC CTG TGT CTG AGA ACG T-3′; reverse: 5′-GAG TAG GGC TCA GCG CAT AAA TA-3′; probe: 5′-CTG AGT CCA CCT GAC ACC CCA CAC C-3′); HRV (forward: 5′-CCT CCG GCC CCT GAA T-3′; reverse: 5′-GGT CCC ATC CCG CAA TT-3′, probe: 5′-CTA ACC TTA AAC CTG CAG CCA-3′). Housekeeping gene GAPDH (4352934E, Applied Biosystems, Foster City, CA) was evaluated as an internal positive control. The comparative cycle of threshold (ΔΔCt) method was used to demonstrate the relative levels of target genes.

### ELISA

Human IFN-λ1 protein levels in cell culture supernatants were determined by using a human IFN-λ1 DuoSet ELISA Development Kit (DY7246, R&D Systems, Minneapolis, MN).

### Statistical analysis

Data are presented as means ± SEM. One-way analysis of variance (ANOVA) was used for multiple comparisons, and a Tukey’s post hoc test was applied where appropriate. Student’s *t* test was used when only two groups were compared. A *p* value < 0.05 was considered significant.

## Results

### Trehalose inhibits IFN-λ1 expression and promotes HRV-16 replication in normal human primary airway epithelial cells

To determine whether trehalose regulates the airway epithelial anti-viral responses, we measured IFN-λ1 expression and HRV load in NHTE cells after treatment with or without trehalose and HRV-16 for 6 and 24 h. The 6 and 24 h time points post infection were chosen based on our preliminary time-course (6, 24 and 48 h) optimization experiments where cells were infected with HRV-16 at the dose of 10^4^ TCID_50_/well. We found that HRV-16 levels were increased at 6 h, and maintained at 24 h, but not at 48 h.

**Fig 1:**
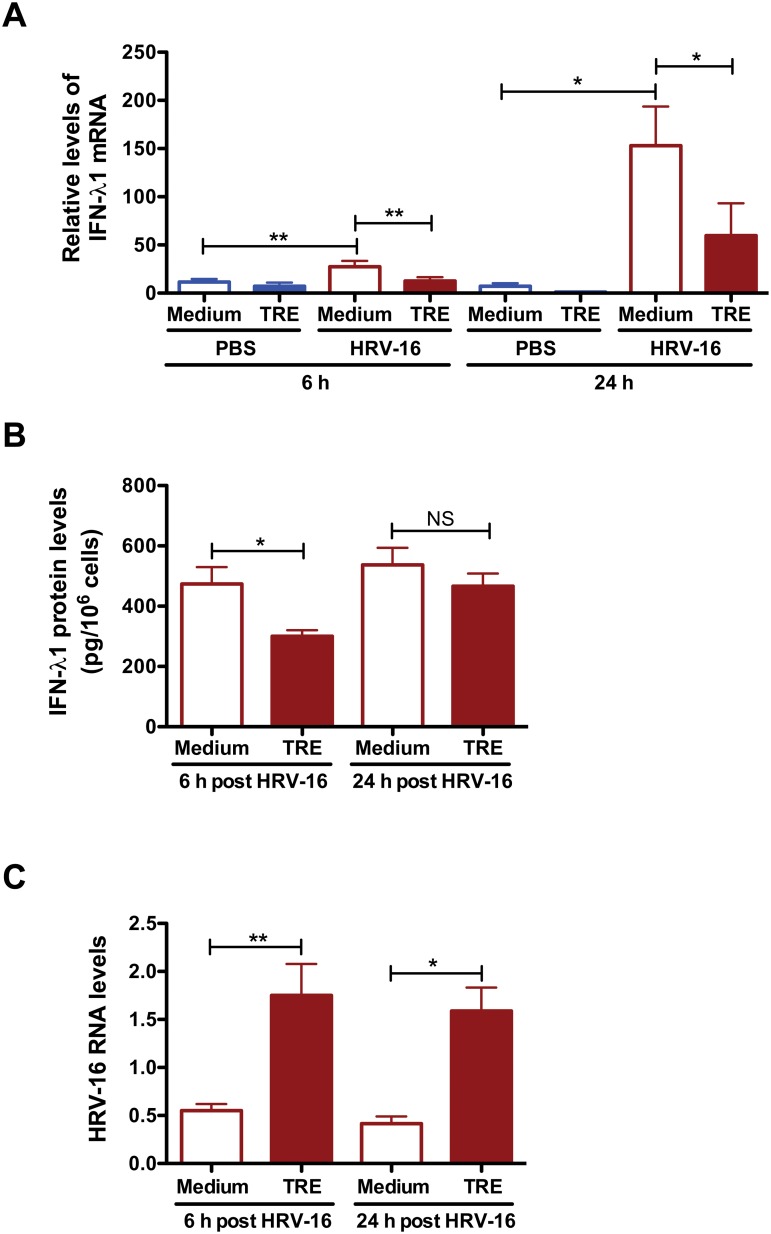
Trehalose inhibits IFN-λ1 expression and promotes HRV-16 replication in normal human primary airway epithelial cells. Normal human tracheobronchial epithelial cells were treated with medium or trehalose (TRE, 100 mM) for 48 h and then infected with HRV-16 (10^4^ TCID_50_/well) for 2 h. After removing the free viruses, cells were incubated with medium or trehalose for additional 6 and 24 h. IFN-λ1 mRNA levels (A) and IFN-λ1 protein levels (B) were assessed by quantitative real-time RT-PCR and ELISA, respectively. HRV-16 RNA levels (C) were examined by quantitative real-time RT-PCR. Data are presented as mean ± SEM (n = 5 independent experiments). NS, not significant; *, p<0.05; **, p<0.01.

HRV-16 infection alone significantly increased IFN-λ1 mRNA levels as compare to cells with medium/PBS treatment at both 6 and 24 h (Fig 1A). Trehalose treatment notably decreased HRV-induced IFN-λ1 mRNA expression compared with medium-treated and HRV-infected cells at both 6 and 24 h. IFN-λ1 protein was undetectable by ELISA in non-infected cells. HRV-induced IFN-λ1 protein secretion was suppressed by trehalose treatment, particularly at 6 h (Fig 1B). To examine if trehalose affects airway epithelial HRV load, we quantified HRV-16 RNA levels in HRV-infected cells. Trehalose treatment significantly increased intracellular HRV-16 RNA levels at 6 h, and maintained at 24 h (Fig 1C).

Taken together, our data suggests that trehalose significantly impairs anti-viral IFN-λ1 expression and promotes HRV-16 infection in normal human primary airway epithelial cells.

### Trehalose induces autophagy in normal human primary airway epithelial cells

**Fig 2:**
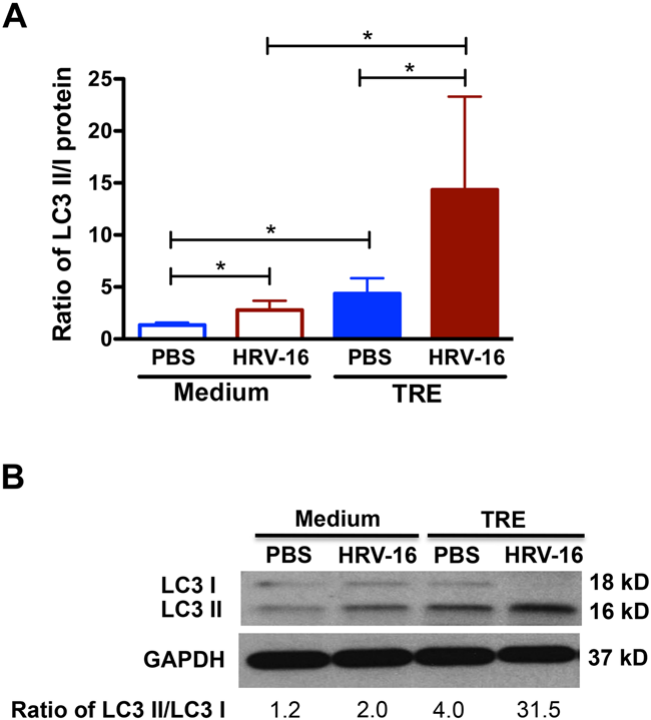
Trehalose induces autophagy in normal human primary airway epithelial cells. Normal human tracheobronchial epithelial cells were treated with medium or trehalose (TRE, 100 mM) for 48 h and then infected with HRV-16 (10^4^ TCID_50_/well) for 2 h. After removing the free viruses, cells were incubated with medium or trehalose for additional 6 h. Protein levels of LC3 I and LC3 II were examined by Western blot analysis with GAPDH protein used as loading control. Data are expressed as mean ± SEM (A) (n = 5 independent experiments, * p<0.05). A representative Western blot picture of LC3 I and LC3 II (B) was shown from 5 independent experiments.

As the 6 h post HRV-16 infection presented the most significant changes of IFN-λ1 expression and viral load with trehalose treatment, we focused on this early time-point to examine the induction of autophagy by trehalose in normal human primary airway epithelial cells. We measured LC3 I and LC3 II protein levels in NHTE cells after treatment with or without trehalose and HRV-16 for 6 h. Without HRV-16 infection, trehalose treatment notably increased accumulation of LC3 II protein compared with medium control (Fig 2). In line with our previous finding that HRV-16 induces autophagy in a human NCI-H292 lung epithelial cell line [32], HRV-16 infection significantly increased the ratio of LC3 II/LC3 I protein in NHTE cells. Trehalose treatment robustly increased LC3 II protein levels following HRV-16 infection as compared to medium-treated and HRV-infected cells.

**Fig 3:**
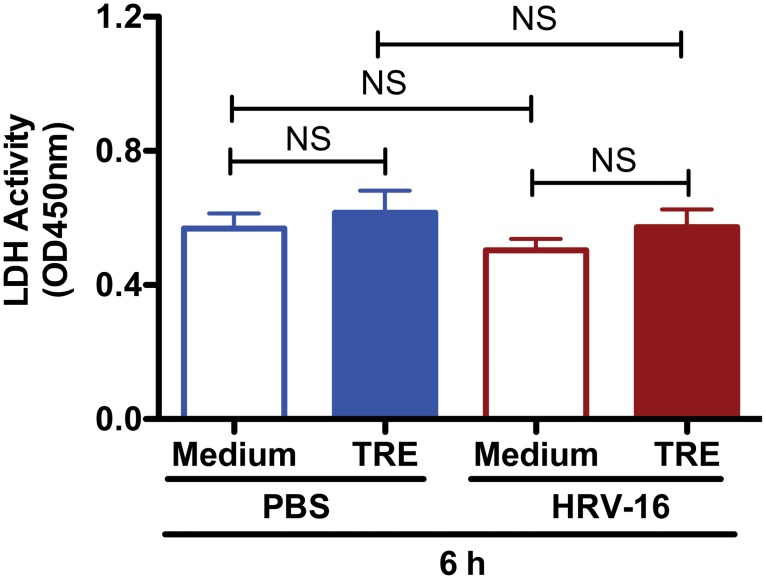
Trehalose exhibits minimal cytotoxic effect in normal human primary airway epithelial cells. Normal human tracheobronchial epithelial cells were treated with medium or trehalose (TRE, 100 mM) for 48 h and then infected with HRV-16 (10^4^ TCID_50_/well) for 2 h. After removing the free viruses, cells were incubated with medium or trehalose for additional 6 h. The cytotoxic effect was assessed by measuring lactate dehydrogenase (LDH) activity in cell culture supernatants. Data are presented as mean ± SEM (n = 5 independent experiments). NS, not significant.

To evaluate the potential cytotoxic effects of trehalose and HRV-16 infection that may affect autophagy in NHTE cells, LDH release was measured in the cell supernatants from the same experiments. No significant increase of LDH activity was observed in cells after treatment with trehalose, HRV-16 or both for 6 h (Fig 3).

All these data suggest that trehalose can efficiently activate the autophagic pathway with minimal cytotoxicity in normal human primary airway epithelial cells.

### Inhibition of the autophagic pathway in normal human primary airway epithelial cells rescues the impaired IFN-λ1 expression by trehalose and subsequently reduces HRV-16 load

**Fig 4:**
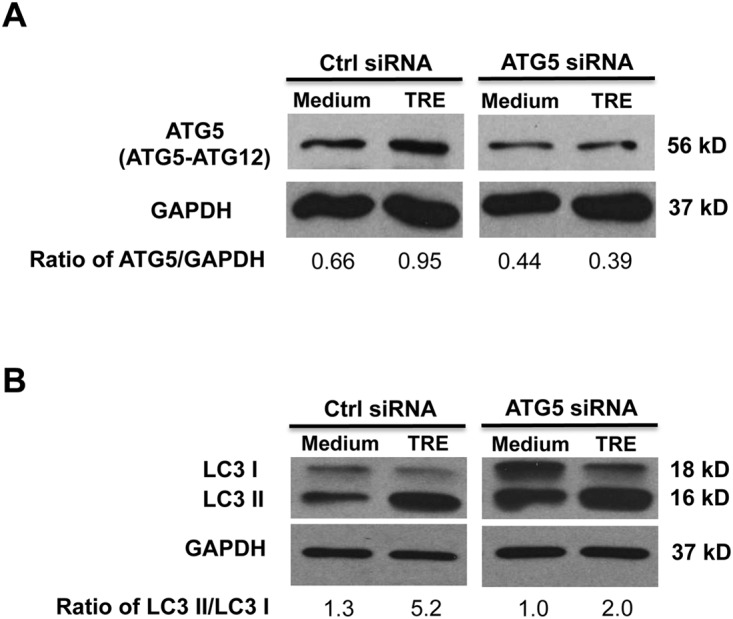
Knockdown of autophagy-related gene 5 (ATG5) inhibits trehalose-induced autophagy in normal human primary airway epithelial cells. Normal human tracheobronchial epithelial cells were transfected with Naito1 chimera RNAi (control siRNA) or ATG5 chimera siRNA (ATG5 siRNA). Twenty-four hours after siRNA transfection, cells were treated with medium or trehalose (TRE, 100 mM) for 48 h. ATG5 protein (A) and LC3 I and LC3 II proteins (B) were examined by Western blot analysis with GAPDH protein used as loading control. The representative Western blot picture was shown from 2 independent experiments with each being performed in triplicate wells.

To determine whether trehalose-induced autophagy contributes to the down-regulation of IFN-λ1 upon HRV-16 infection, ATG5 was knocked down by using target-specific chimera RNA interference [33–35]. Control siRNA- or ATG5 siRNA-transfected NHTE cells were infected with HRV-16 or PBS for 6 h to examine whether ATG5 knockdown alters IFN-λ1 expression and viral load. Western blot analysis confirmed ATG5 protein reduction by ATG5 siRNA in both medium- and trehalose-treated cells (Fig 4A). Intriguingly, ATG5 siRNA was shown to increase both LC3 I and LC3 II basal protein levels although an expected reduction in the ratio of LC3 II/LC3 I protein was observed compared with control siRNA (Fig 4B). Similar results were recently reported in fibroblasts transfected with conventional siRNAs against ATG5 [36]. Trehalose treatment in control siRNA-treated cells markedly increased (4-fold) the ratio of LC3 II/LC3 I protein compared with medium control. However, the conversion of LC3 I into LC3 II after trehalose treatment was decreased by 50% following ATG5 knockdown.

**Fig 5:**
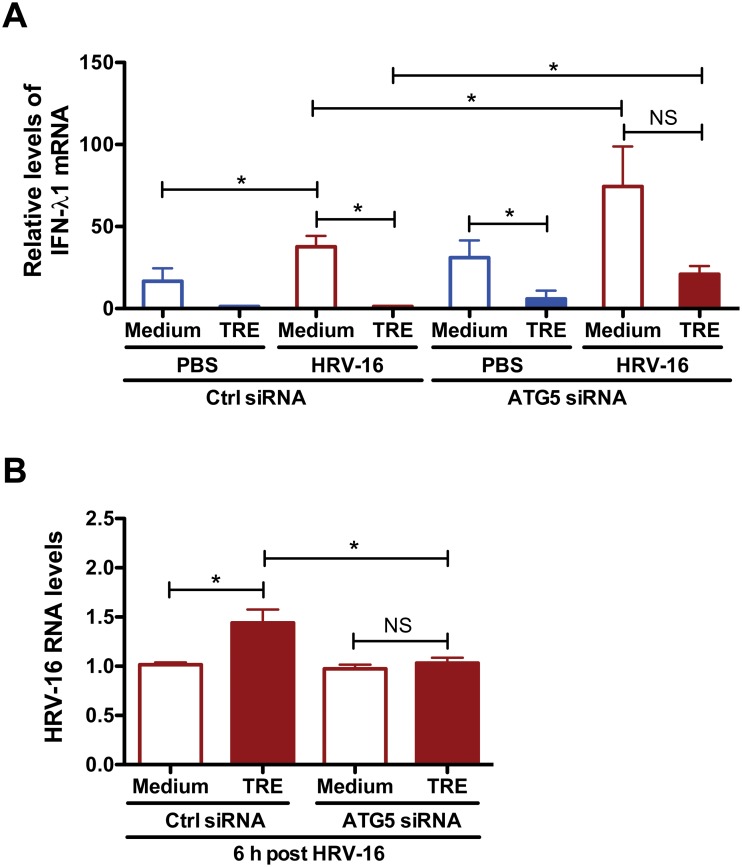
Inhibition of autophagy rescues the impaired IFN-λ1 expression by trehalose and subsequently reduces HRV-16 load in normal human primary airway epithelial cells. Normal human tracheobronchial epithelial cells were transfected with Naito1 chimera RNAi (control siRNA) or ATG5 chimera siRNA (ATG5 siRNA). Twenty-four hours after siRNA transfection, cells were treated with medium or trehalose (TRE, 100 mM) for 48 h and then infected with HRV-16 (10^4^ TCID_50_/well) for 2 h. After removing the free viruses, cells were incubated with medium or trehalose for additional 6 h. The expression of IFN-λ1 mRNA (A) and viral RNA levels (B) were quantified by quantitative real-time RT-PCR. Data are presented as mean ± SEM (n = 2 independent experiments with each being performed in triplicate wells). NS, not significant; *, p<0.05.

In keeping with above data, trehalose treatment significantly inhibited HRV-induced IFN-λ1 mRNA expression in both control siRNA- and ATG5 siRNA-treated cells (Fig 5A). Importantly, HRV-induced IFN-λ1 mRNA levels were significantly higher in ATG5 siRNA-treated cells vs. control siRNA-treated cells. Lastly, ATG5 knockdown prevented the increase of intracellular HRV-16 RNA levels after trehalose treatment (Fig 5B).

Collectively, these results indicate that the impaired IFN-λ1 expression and enhanced HRV replication after trehalose treatment is dependent on induction of autophagy in normal human primary airway epithelial cells.

### ATG5 protein interacts with RIG-I and IPS-1 in normal human primary airway epithelial cells

**Fig 6:**
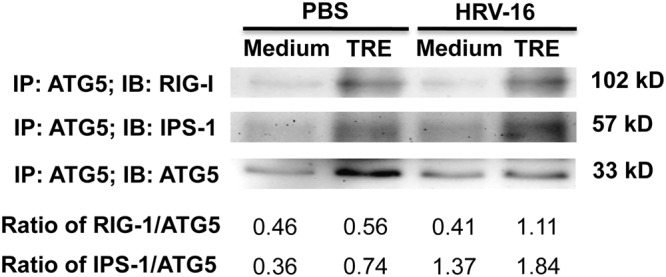
Autophagy-related gene 5 (ATG5) protein interacts with retinoic acid-inducible gene I (RIG-I) and IFN-β promoter stimulator 1 (IPS-1) in normal human primary airway epithelial cells. Normal human tracheobronchial epithelial cells were treated with medium or trehalose (TRE, 100 mM) for 48 h and then infected with HRV-16 (10^4^ TCID_50_/well) for 2 h. After removing the free viruses, cells were incubated with medium or trehalose for additional 6 h. Pre-cleared cell lysates were incubated with a mouse anti-human ATG5 antibody, and immunoprecipitated proteins were separated on 10% SDS—PAGE for immunoblotting of RIG-I, IPS-1 and ATG5. The representative Western blot picture was shown from 2 independent experiments with each being performed in triplicate wells.

To uncover the potential molecular mechanisms involved in autophagy-mediated suppression of IFN-λ1, the interaction of ATG5 protein with RIG-I and IPS-1 was examined by ATG5 pull-down, followed by immunoblotting of RIG-I and IPS-1 in NHTE cells after treatment with or without trehalose and HRV-16 for 6 h. ATG5 protein was constitutively expressed at a low level in cultured NHTE cells. RIG-I and IPS-1 were co-immunoprecipitated with ATG5 protein (Fig 6). A stronger interaction of RIG-I and IPS-1 with ATG5 protein was observed in trehalose-treated cells, especially with HRV-16 infection. These observations indicate that ATG5 may interact with RIG-I and IPS-1 upon induction of autophagy in normal human primary airway epithelial cells.

## Discussion

This is the first study to provide direct evidence that autophagy impairs the anti-viral interferon response and facilitates HRV infection in human airway epithelial cells. We discovered that trehalose-induced autophagy directly inhibits IFN-λ1 expression and promotes HRV-16 infection in normal human primary airway epithelial cells.

Despite overwhelming evidence linking HRV infections to exacerbations of asthma and other lung diseases, the mechanisms of frequent airway HRV infections are poorly understood. Impaired IFN-λ1 induction in HRV-infected asthmatic airway epithelial cells may in part explain the increased viral infection during acute exacerbations [1–3], but the underlying mechanisms have not been well elucidated. Autophagy has been proposed as a novel mechanism in response to viral infections. Although autophagy is increased in asthmatic airway epithelial cells [13, 14], its role in the anti-viral interferon response remains uncertain. Our previous publication [32] indicates that interleukin-1 receptor-associated kinase M (IRAK-M), a negative regulator of innate immunity, promotes lung epithelial HRV-16 infection in part through the activation of autophagic pathway. However, whether autophagy directly impairs the anti-viral interferon response has not been addressed. Autophagy can be induced by various physiological, pathological and pharmacological factors. In the current study, we used trehalose, a natural compound, to induce autophagy as it exhibits minimal cytotoxic effect in a variety of mammalian cells [24, 25]. Indeed, trehalose did not show any cytotoxicity in normal human primary airway epithelial cells that may compromise our conclusion about the role of autophagy in host defense against HRV infection. Our data clearly reveal that trehalose-induced autophagy directly inhibits the expression of IFN-λ1 and promotes viral replication in HRV-16-infected normal human primary airway epithelial cells.

For the first time, we have applied chimera RNAi, a novel mammalian gene-silencing tool, in human primary airway epithelial cells. ATG5 chimera siRNA successfully reduces ATG5 protein and leads to inhibition of autophagy reflected by a reduction in the ratio of LC3 II/LC3 I protein in NHTE cells. Blocking trehalose-induced autophagy via ATG5 knockdown effectively rescues the impairment of IFN-λ1 expression after trehalose treatment and subsequently reduces HRV-16 load. Our research findings suggest induction of autophagy as a novel mechanism to hinder host anti-viral defense against respiratory viral (e.g., HRV-16) infections in the lung. Of note, we have observed that control chimera siRNA alone slightly increased LC3 II expression in NHTE cells. This data is similar to a previous report that the transfection reagent (Lipofectamine 2000) and negative control siRNA complex could increase autophagosome formation [37]. Since activation of airway epithelial autophagy promotes HRV-16 replication [32], the increase of the basal autophagy levels caused by chimera siRNA transfection may be partially responsible for the smaller difference of HRV-16 load at 6 h between medium and trehalose treatment groups (Fig 5B) as compared to cells without chimera siRNA transfection (Fig 1C).

How trehalose-mediated autophagy impairs the anti-viral interferon response in airway epithelial cells remains to be elucidated. Upon RNA viral infections, RIG-I recognizes viral RNA in the cytoplasm of infected cells and then binds to IPS-1 to induce the production of interferons and host anti-viral defense [38, 39]. A recent study has demonstrated that IPS-1 is essential for type III IFN production by hepatocytes and dendritic cells in response to hepatitis C virus infection [40]. Interestingly, ATG5-ATG12 conjugate negatively regulates type I IFN production by direct association with RIG-I and IPS-1 through the caspase recruitment domains [18–21]. As trehalose increases ATG5-ATG12 conjugate, we sought to examine if ATG5 interacts with RIG-I and IPS-1 in human airway epithelial cells. Our data suggests that ATG5 protein interacts with RIG-I and IPS-1 in NHTE cells, especially when the autophagic pathway is activated by trehalose and HRV infection. Such an interaction may be responsible for the suppression of type III IFN-λ1 expression and the subsequent increase of HRV replication in normal human primary airway epithelial cells. Future studies are warranted to clarify the detailed molecular mechanisms by which the interaction of ATG5 with RIG-I and IPS-1 in human airway epithelial cells inhibits IFN-λ1 expression following HRV infection.

In summary, our research findings indicate that induction of autophagy directly impairs the expression of anti-viral type III IFN-λ1 and enhances HRV-16 infection in normal human primary airway epithelial cells. A better understanding of the role of autophagy in airway epithelial defense against HRV infection may lead to novel interventions to attenuate viral infections during acute exacerbations of asthma and other chronic lung diseases.
